# Ancestry of the Brazilian *TP53* c.1010G>A (p.Arg337His, R337H) Founder Mutation: Clues from Haplotyping of Short Tandem Repeats on Chromosome 17p

**DOI:** 10.1371/journal.pone.0143262

**Published:** 2015-11-30

**Authors:** Diego Davila Paskulin, Juliana Giacomazzi, Maria Isabel Achatz, Sandra Costa, Rui Manoel Reis, Pierre Hainaut, Sidney Emanuel Batista dos Santos, Patricia Ashton-Prolla

**Affiliations:** 1 Post-Graduate Program, Genetics and Molecular Biology, Federal University of Rio Grande do Sul, Porto Alegre, Brazil; 2 Genomic Medicine Laboratory, Experimental Research Center, Hospital de Clinicas de Porto Alegre, Porto Alegre, Brazil; 3 Department of Oncogenetics, AC Camargo Cancer Center, Sao Paulo, Brazil; 4 Life and Health Sciences Research Institute, University of Minho, Braga, Portugal; ICVS/3B's-PT Government Associate Laboratory, Braga/Guimarães, Portugal; 5 Molecular Oncology Research Center, Barretos Cancer Hospital, Barretos, São Paulo, Brazil; 6 International Prevention Research Institute, Lyon and Institut Albert Bonniot/INSERM 823, Grenoble, France; 7 Human and Medical Genetics Laboratory, Biosciences Institute, Federal University of Pará, Belém, Brazil; 8 Medical Genetics Service), Hospital de Clinicas de Porto Alegre, Porto Alegre, Brazil; University of Texas MD Anderson Cancer Center, UNITED STATES

## Abstract

Rare germline mutations in *TP53* (17p13.1) cause a highly penetrant predisposition to a specific spectrum of early cancers, defining the Li-Fraumeni Syndrome (LFS). A germline mutation at codon 337 (p.Arg337His, c1010G>A) is found in about 0.3% of the population of Southern Brazil. This mutation is associated with partially penetrant LFS traits and is found in the germline of patients with early cancers of the LFS spectrum unselected for familial history. To characterize the extended haplotypes carrying the mutation, we have genotyped 9 short tandem repeats on chromosome 17p in 12 trios of Brazilian p.Arg337His carriers. Results confirm that all share a common ancestor haplotype of Caucasian/Portuguese-Iberic origin, distant in about 72–84 generations (2000 years assuming a 25 years intergenerational distance) and thus pre-dating European migration to Brazil. So far, the founder p.Arg337His haplotype has not been detected outside Brazil, with the exception of two residents of Portugal, one of them of Brazilian origin. On the other hand, increased meiotic recombination in p.Arg337His carriers may account for higher than expected haplotype diversity. Further studies comparing haplotypes in populations of Brazil and of other areas of Portuguese migration are needed to understand the historical context of this mutation in Brazil.

## Introduction

A germline *TP53* mutation in codon 337 (c.1010G>A; p.Arg337His) is present at a high frequency (0.3%) and represents a significant cancer risk factor in the population of Southern Brazil [1–4]. This mutation predisposes to multiple and early cancers that characterize the tumor spectrum of Li-Fraumeni Syndrome (LFS) and its variant Li-Fraumeni-Like syndrome (LFL) [5–8]. Initially described in association only with adrenocortical carcinoma (ADC) in Brazilian children with no familial cancer history (Ribeiro *et al*., 2001), p.Arg337His was subsequently identified in the germline of individuals with cancer predisposition matching LFS/LFL criteria, including early onset breast cancer, soft tissue sarcoma, choroid plexus carcinoma, and phyllodes breast tumors [9–14]. To date, germline p.Arg337His has been reported in over 50 cancer-prone Brazilian families and in several hundred of Brazilian cancer patients with no apparent familial history of cancer. To our knowledge, only 2 instances of the germline mutation have been reported in patients with no known connection with Brazil: a family of Portuguese origin matching LFS criteria, with a 8-years old female proband diagnosed with ADC in France [15,16], and a family of German origin that does not match LFS/LFL criteria, with a 71 year-old proband diagnosed with ADC [17,18].

Despite initial reports suggesting that the mutation may have arisen independently in children with apparently sporadic ADC, in-depth analysis of 29 *TP53* tag single nucleotide polymorphisms in 48 unrelated subjects (45 Brazilians, and 3 Portuguese) demonstrated that the mutation was present on an identical founder haplotype of Caucasian origin, suggesting that the p.Arg337His mutation had probably arisen in an individual of European ancestry [15]. Interestingly, this founder haplotype was not detected in the patient from Germany [17,18] suggesting that in this patient the mutation resulted from an independent event.

In the present study, we have further assessed the ancestry of the founder allele in Brazilian subjects by haplotyping nine short tandem repeats (STRs) spanning 8Mb on the entire short arm of chromosome 17 and flanking the *TP53* gene at 17p13.1. We estimated mutation age and compared haplotypes in carriers with those of reference populations from Brazil, Portugal, Africa, as well as Amerindians in order to infer the population origin of the founder allele.

## Materials and Methods

### Participants

Patients and cancer-unaffected relatives carrying *TP53* p.Arg337His were recruited for the study after signature of informed consent as this study was approved by the Research Ethics Committee of the Research and Postgraduate Studies Group from Hospital de Clinicas de Porto Alegre (CEP-GPPG-HCPA) under protocol number #09–430. DNA samples from 12 apparently unrelated familial trios (father-mother-child) were included. Of these, nine were recruited from Hospital de Clinicas de Porto Alegre (city of Porto Alegre, in the State of Rio Grande do Sul, Brazil) and three were recruited from AC Camargo Cancer Center (city of São Paulo, State of São Paulo, Brazil).

To assess the most likely populational origin of the founder haplotype, we analyzed unidentified DNA samples from different series of cancer-unaffected individuals, which had been previously obtained for populational genetic analyses in different studies after ethical approval. These include a series of 250 cancer-unaffected individuals from Southern Brazil (population-based sample), 270 cancer-unaffected and otherwise asymptomatic subjects from Northern Portugal [19,20], 120 asymptomatic subjects from the Sub-Saharan region of Africa [21,22] and 100 individuals from eight Amerindian tribes belonging to four linguistic groups of the Brazilian Amazon Region [23].

### Genotyping

**Fig 1:**
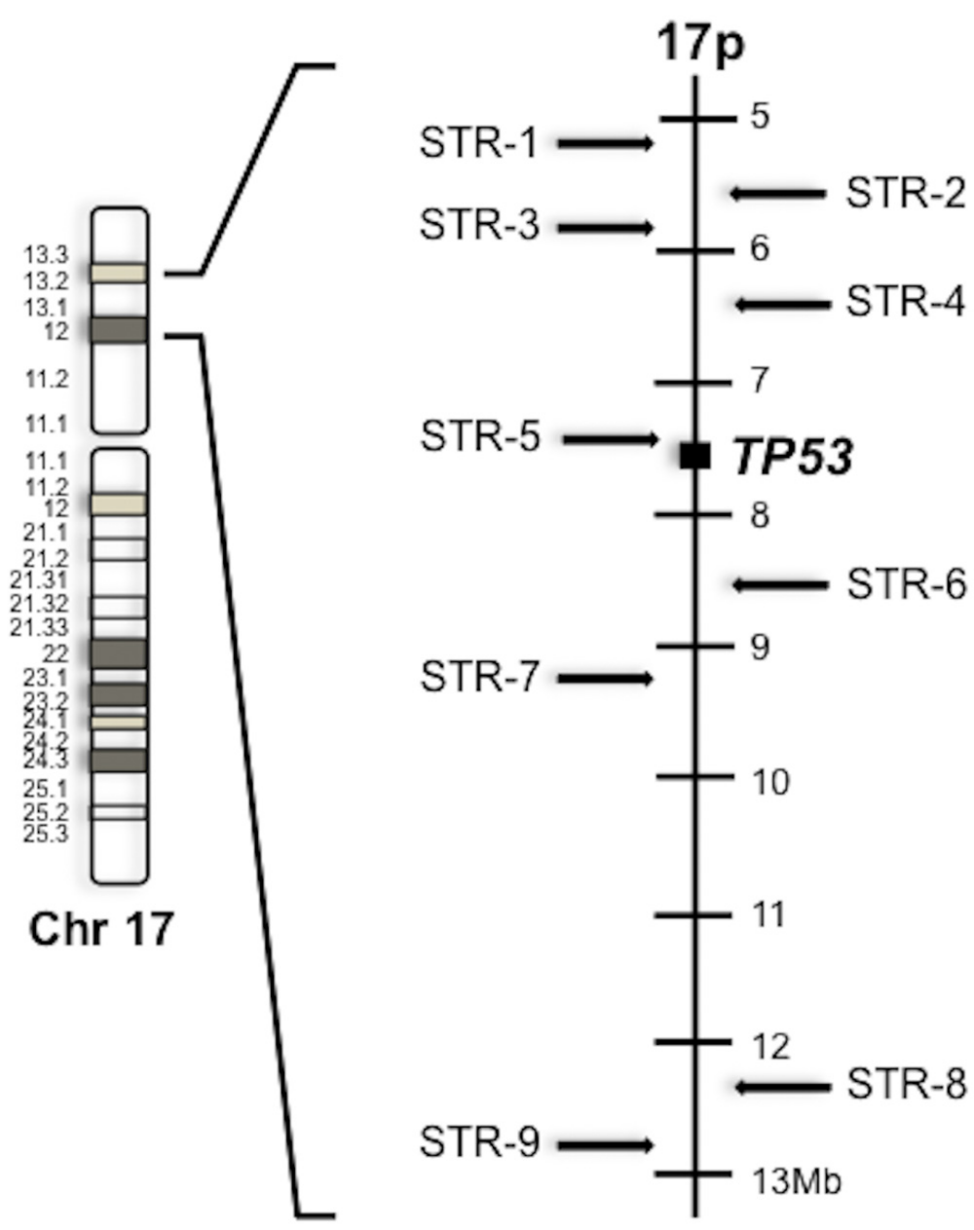
Short Tandem Repeats position used for haplotype analysis and their respective position in chromosome 17p.

Genomic DNA was extracted from peripheral blood leukocytes using the Ilustra blood genomic Prep Mini spin Kit (GE Healthcare, Piscataway, NJ, USA) as described by the manufacturer. DNA concentration was measured with Nano-Drop 1000 (Thermo Fisher Scientific, Waltham, MA, USA) and diluted to a final concentration of 10 ng/μl. Direct sequencing of *TP53* exons 2 to 11 and flanking splice site junctions was performed in all members of the trios as previously described [24]. For haplotype analysis, we selected nine short tandem repeat (STRs) markers spanning a region of 8Mb extending on both sides of the *TP53* gene at 17p13 (Fig 1). STR1 to STR9 markers were PCR amplified using fluorescently end-labeled primers (Table 1), analyzed on an ABI3130 DNA analyzer and scored using ABI GeneScan Analysis Software (Thermo Fisher Scientific, Waltham,MA, USA).

**Table 1:** Primers and Short Tandem Repeats genomic position.

STR	Repeat	Genomic Position	Primers
STR-1	(AATA)n	5235917	F - 6FAM 5′TTAGGAAAGGGTTGCCAGATTA and R - 5′CAAACTTCCCTTGACCATCACT
STR2	(ACAC)n	5676955	F - 5′ACTCCAGCATGGGTAACAGAGT and R - 6FAM 5′TATCCCAACCATATCCTCCAAA
STR-3	(TGAGC)n	5913810	F - NED 5′ACATACGAAGCATCCAGTGAAG and R - 5′AGGATTAGCATCAGGTTTCCAG
STR-4	(TGTTT)n	6154523	F - NED 5′TGACTTTTGGGACTTTGTGTGT and R - 5′GGAGACAGAGGTTGCAGTGAG
STR-5	(AAAC)n	7534398	F - 5′GGACAGAGCAAAACTCCATCTC and R - PET 5′ATTTCTGGGAGGACACAACAAG
STR-6	(ATAG)n	8600727	F - VIC 5′ACACGGAACGGAATATCCTACA and R - 5′GCACCTGTACTCCCAGCTACTT
STR-7	(ATAA)n	9248467	F - PET 5′ATATGGATGGGAGGACAAGAGA and R - 5′GGATGGATGGATAGGCAGATAG
STR-8	(GCAC)n	12351781	F - 6FAM 5′CACCACAAACTTTATTGCTTCG and R - 5′CTAAATCTGCAAGTCCCCTTTG
STR-9	(AGC)n	12800569	F - VIC 5′GGATGAAGTAAGGGCAATGAAC and 9R - 5′GCTTGGACGACAGAGTGAGAC

### Age estimation and ancestry of p.Arg337His

To estimate the age (in generations) of the *TP53* p.Arg337His mutation we applied two calculation methods using DMLE+2.3 [25] and ESTIAGE [26] softwares. DMLE+2.3 allows Bayesian inference of the generation distance of the most plausible common ancestor based on the observed linkage disequilibrium at multiple genetic markers in carriers and unrelated non-carriers. This method uses a Markov Chain Monte Carlo algorithm taking into account the proportion of disease bearing chromosomes carrying the *TP53* p.Arg337His mutation (0.015%) [2,3], the distances between the markers and the mutation site relative to the markers using HG19 sequence and 1cM = 1Mb, and an estimated annual Brazilian population growth rate of 9.5%, based on the mean of the Brazilian growth rate for 300 years (45% including reproductive growth but also including the distinct waves of immigration) and the European growth rate for 1700 years (2.5%). ESTIAGE is a likelihood-based method that uses multilocus marker data from patients carrying the same mutation, assuming that they descended from a common ancestor who introduced the mutation. An estimate of the number of generations since the most recent common ancestor is obtained from the size of the haplotype shared by the individuals on each side of the disease locus. The method uses haplotype information in patients carrying the mutation and in control subjects to identify the most likely positions of recombination events on the ancestral haplotype. A 25-year intergeneration interval was used for both methods with a mutational rate of 10^−4^ for the nine STRs analyzed.

After genotypes were established, haplotype frequencies were estimated using the ARLEQUIN v3.5 software [27] in 500 chromosomes from unrelated control individuals from Porto Alegre, 540 chromosomes from unrelated Portuguese individuals, 240 chromosomes from unrelated African individuals and 200 chromosomes from Native American individuals. ARLEQUIN v3.5 was also used to compare the ancestral haplotype in which the p.Arg337His mutation is present with the constructed parental population haplotypes, assuming that the most likely parental population would present the least average pairwise difference with the ancestral haplotype in which the p.Arg337His mutation is present [28,29].

## Results

### Age estimation of the *TP53* p.Arg337His mutation

**Fig 2:**
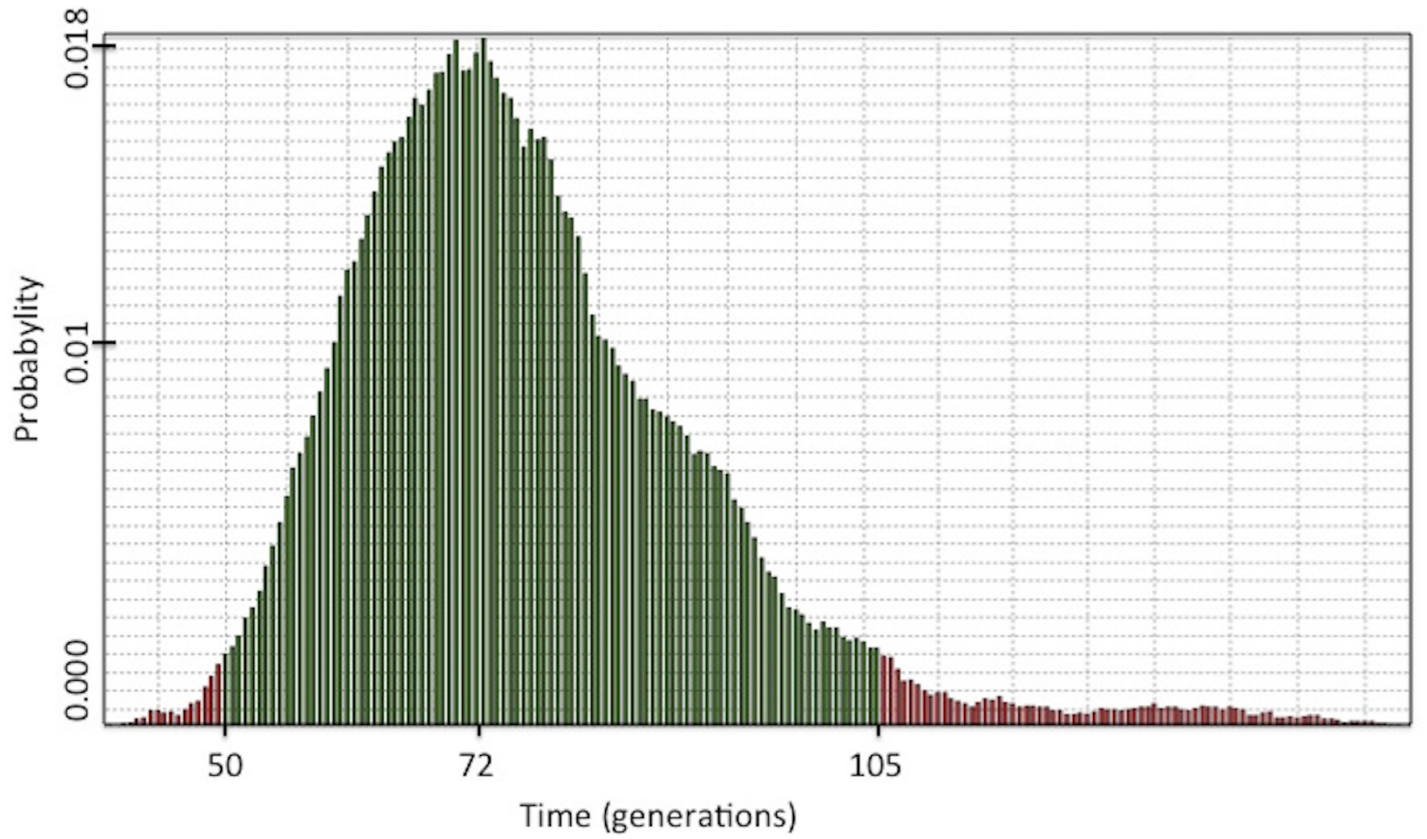
Age estimation for the p.Arg337His mutation as assessed by DMLE+2.3. Green lines show 95% CIs.

To establish the correct haplotype in which the p.Arg337His allele is present, we used genotype data from the nine STRs analyzed in twelve trios. Observed haplotypes of individuals carrying the p.Arg337His allele are shown in Table 2. Analysis of the nine STR markers in twelve trios using the ESTIAGE software revealed that all extended haplotypes carrying the p.Arg337His mutation included in this study derive from a common ancestor haplotype which was introduced in the population 84 generations ago (95% CI: 54–135). For estimating the generation distance using the DMLE+2.3 software, the haplotype frequencies from the Portuguese control population were constructed using ARLEQUIN as a reference. Analysis of linkage disequilibrium in carriers and non-carriers estimated that the p.Arg337His mutation was present on a haplotype that might have been introduced 72 generations ago (95% CI 50–105) (Fig 2). Based on these two approaches and using a pre-established 25-year intergeneration interval, the age of the *TP53* p.Arg337His mutation appears to be of approximately of 2000 years.

**Table 2:** *TP53* p.R337H mutation carrier’s haplotypes.

	STR-1	STR-2	STR-3	STR-4	STR-5	R337H	STR-6	STR-7	STR-8	STR-9
POA-1	270	181	138	225	**224**	**337H**	**262**	**167**	**112**	191
POA-2	248	**189**	**108**	**245**	**224**	**337H**	**262**	**167**	**112**	203
POA-3	248	**189**	**108**	**245**	**224**	**337H**	**262**	**167**	108	191
POA-4	248	**189**	**108**	**245**	**224**	**337H**	**262**	163	108	203
POA-5	270	**189**	**108**	**245**	**224**	**337H**	270	175	108	191
POA-6	**274**	**189**	**108**	**245**	**224**	**337H**	**262**	171	**112**	191
POA-7	**274**	**189**	**108**	**245**	**224**	**337H**	**262**	**167**	**112**	191
POA-8	**274**	**189**	**108**	**245**	**224**	**337H**	282	175	**112**	191
POA-9	248	**189**	143	**245**	**224**	**337H**	**262**	**167**	108	191
SP-1	**274**	**189**	**108**	**245**	**224**	**337H**	**262**	159	108	191
SP-2	248	181	138	235	**224**	**337H**	**262**	**167**	**112**	191
SP-3	270	185	138	**245**	**224**	**337H**	**262**	**167**	**112**	194

Shared haplotypes are highlighted in bold.

### Population origin of the *TP53* p.Arg337His mutation

In order to identify the most likely population origin of the *TP53* p.Arg337His mutation, we genotyped individuals from parental populations that contributed to the current admixed Brazilian population, as described in the methods section. Using ARLEQUIN v3.1 to identify the most likely origin based on the average pairwise differences between the parental population haplotypes and the twelve haplotypes carrying the mutation (Table 3), we observed that the founder p.ArgR377His haplotype had the highest homology with the haplotypes of Portuguese (Average Pairwise Difference = 1.71096, P<0.001) and Brazilian reference population, and the lowest homology with haplotypes of Amerindian and African ancestries. These results reinforce previous indications that the haplotype carrying p.Arg337His is of European/Caucasian origin [15], and suggest a Portuguese-Iberic origin, although subgroups of European ancestry were not analyzed.

**Table 3:** Average Pairwise Differences Among Haplotypes.

	R337H carriers Haplotypes	*P*-Value
Portuguese	1.71096	<0.001
Brazilians	1.75029	<0.001
Amerindians	2.37292	<0.001
Africans	2.76101	<0.001

## Discussion

Analysis of 9 STRs on the short arm of chromosome 17p in 12 unrelated trios from Brazilian families carrying the *TP53* p.Arg337His mutation confirms that all carriers have a common ancestry. Comparing the most plausible ancestor haplotype with those present in populations contributing to the current admixed population of Brazil, the founder haplotype appears to be of European/Portuguese-Iberic origin. Furthermore, estimates of the generation distance to the common ancestor of the 12 trios analyzed suggest that p.Arg337His carriers are separated by about 72–84 generations, representing approximately 2000 years assuming a 25 years intergenerational distance. Consistent estimates were obtained with two different computational approaches to calculate generational distance, using either the DMLE+2.3 (72 generations) and ESTIAGE (84 generations) softwares. It is important to note that current evidence does not show increased recombination rates in carriers of germline *TP53* mutations [29], and although recombination rates are heterogeneous across the genome with higher rates in certain chromosomes (including chromosome 17), a recombination rate up to 20% over baseline would not significantly impact age estimation as calculated here [28,30]. These results confirm, strengthen and extended previous studies [15] demonstrating a founder effect based on the analysis of intragenic *TP53* polymorphism and provide novel information on the diversity of the extended haplotypes that are present in p.Arg337His carriers. The present study differs from Garritano’s in two main aspects. First, Garritano’s paper was focused in confirming whether p.Arg337His is a founder mutation and present in depth *TP53* sequencing data to provide the answer to this question, while our main goal was to determine mutation age and obtain insights about mutation origin. Second, in terms of methodology, Garritano et al analyzed intragenic TP53 single nucleotide polymorphisms, while we focused on STR markers spanning a region of 8Mb downstream and upstream of the TP53 gene. We chose this approach because the STRs used are positioned in non-coding regions of the genome being biologically silent, which enables accumulation of mutations unhindered over the generation, facilitating the identification of molecular variability. Indeed, Garritano et al. suggested that the analysis of an extended haplotype would help to correctly identify the datation of the founder effect demonstrated by their work.

The 12 trios who contributed DNA for this analysis are from families of apparent European origin, established in Southern Brazil since an unknown number of generations. The current Brazilian population is the result of five centuries of admixture between different continental ancestries (Europeans, Africans and Amerindians) [31–33]. Although the majority of the contribution to the current gene pool is European in all Brazilian regions, Amerindian and African ancestries are have a higher representation in the population of Northern and North-Eastern Brazil (about 4 million African individuals are estimated to have been brought to Brazil as slaves between the years 1700 and 1850). In contrast, the population of Southern and Southeastern Brazil shows higher proportions of European ancestries, probably due to waves of immigration between 16th and mid-20th centuries. The first large wave of immigrants (1500 and 1800) was of Portuguese origin (estimated to about 0.5 millions), followed by successive waves of Germans, Italians, Hispanics, Jews and Japanese, together amounting to about 5 million settlers who are the origin of the current population [34]. Presence of p.Arg337His on a haplotype of Portuguese ancestry is consistent with the population structure of Southern Brazil, where carriers and their families originate. The largest known mutation-positive family includes 9 documented generations and originates from an ancestor of Portuguese origin who reportedly settled in the area of São Paulo in the final years of the 17th century [14].

It is estimated that p.Arg337His carriers represent about 0.3% of the population of Southern Brazil, in a large area extending from the States of Rio de Janeiro (RJ) and Minas Gerais (MG) to the North and North-West, to Rio Grande do Sul (RS) to the South, and encompassing also the States of Parana (PR) and Santa Catarina (SC). Overall, the population of these States amounts to about 100 million, suggesting that about 300,000 of today’s Brazilians may be p.Arg337His carriers. These estimates are based on a small prevalence study in the area of Porto Alegre, State of Rio Grande do Sul (RS; 750 subjects, 2 p.Arg337His carriers; Palmero et al., 2009) and on population-based newborn screening in the State of Paraná (171,469 subjects; 461 carriers; Custódio et al., 2012). These numbers, as well as the wide geographic extension of carriers, suggest that Portuguese migrants introduced the mutation in the first decades of colonization, before the spread of Portuguese explorers and settlers in Southern Brazil in the middle of the 17th century. To date, studies on cancer-prone families and on patients with different core tumors of the LFS/LFL syndromes have identified several hundred Brazilian carriers [1,3,11,14,35–37]. There is currently no data on the prevalence of the mutation in other populations, with only 3 documented instances of the mutation in subjects living outside Brazil. Two were probands from LFS/LFL families diagnosed in Portugal [18] and France, [38] and both were found carry to the same mutant *TP53* haplotype observed in Brazilian carriers. The proband detected in Portugal was of Brazilian origin (reverse-migrant to Portugal), whereas the proband tested in France resided in Portugal, with no information available on a possible Brazilian origin. The third reported occurrence of p.Arg337His outside Brazil is a cancer patient with no LFS/LFL history residing in Germany, who carried the mutation on a different *TP53* background than Brazilian subjects [17,18]. This patient had no known Portuguese or Brazilian ancestry and may represent an independent occurrence of the mutation, a finding consistent with the fact that the G>A mutation at position 1100 is located within a CpG site, a sequence context which is intrinsically highly mutable.

The worldwide expansion of Portuguese explorers, merchants and immigrants between the 16th and 17th centuries has served as vector for well-documented founder genetic effects. Machado-Joseph Disease (MLD) is a dominant form of ataxia caused by a mutation originating in Asia dispersed around the world by Portuguese merchants, sailors and travellers [39]. The *BRCA2* c.156_157insAlu mutation, which accounts for 37.9% of all mutations in families with hereditary breast and ovarian cancer (HBOC) in Portugal, is carried by a Portuguese haplotype occasionally found in countries of Portuguese immigration. In Brazil, this mutation has been observed in 3/168 unrelated HBOC patients from RJ in Brazil, but in none of 144 HBOC probands from SP and RS, Brazil [40]. The estimated age this mutation is 558 ± 215 years, consistent with a temporal origin around the time of the first waves of Portuguese migration. Another Portuguese founder mutation has been observed in *MSH2* (c.388-389 delCA) associated with Lynch syndrome in families from Northern Portugal. The estimated age of this mutation is less than 300 years ago and it has also been reported in Brazilian families with Lynch syndrome [41,42]. In addition, Portuguese founder mutations have been observed among Brazilian patients with other genetic disorders, but different from the *TP53* p.Arg337His mutation, they continue to occur at relatively high frequency in their populations of origin [43,44].

**Fig 3:**
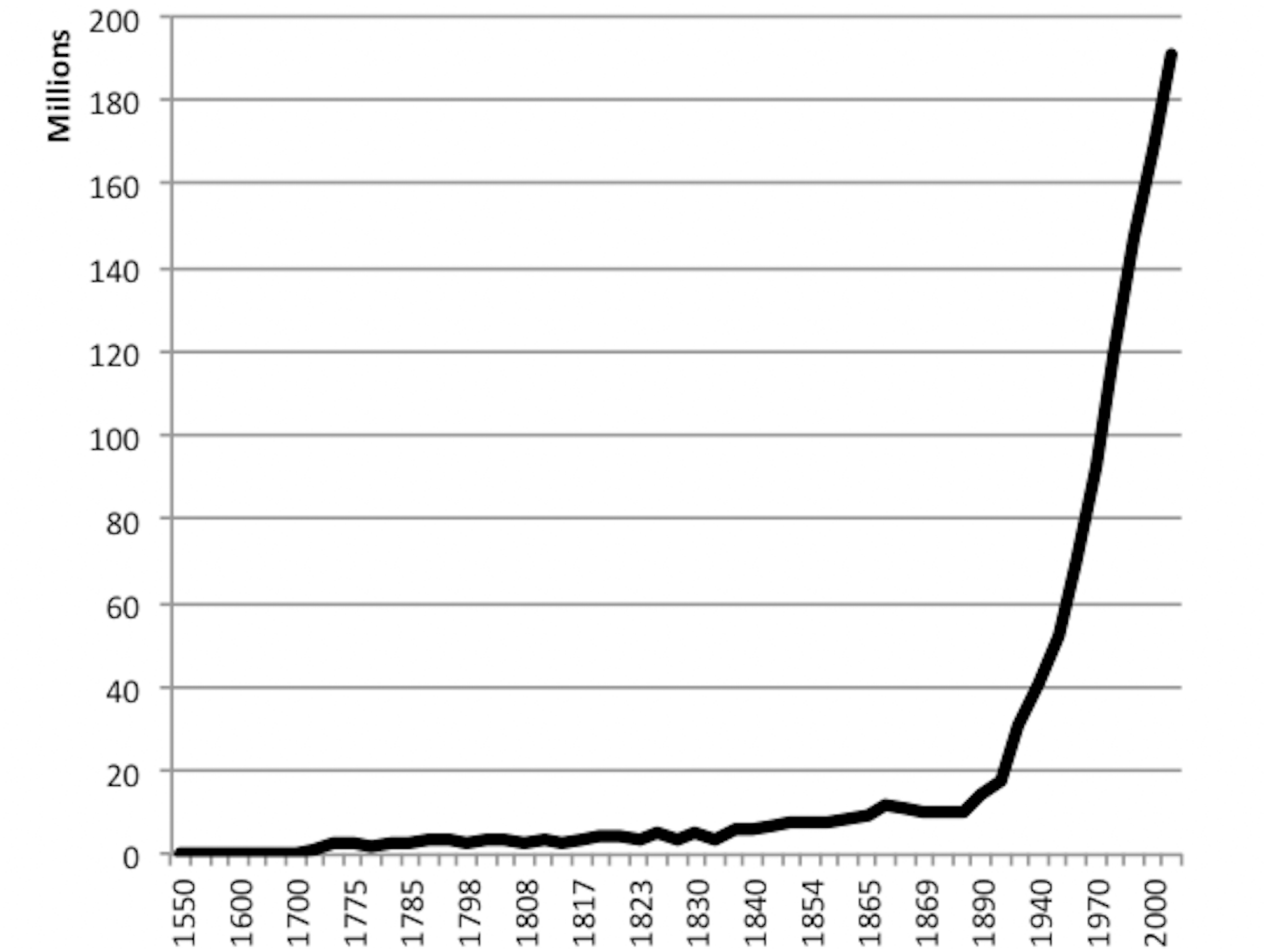
Brazilian Population Growth between 1550–2000.

Our estimate for the age of the mutation should be considered with caution. First, because absolutely precise population growth rates for the last 500 years in Brazil cannot be established, and we have used, as have many others, an estimate of this growth (Fig 3). Second, an apparent generation distance of 72–84 generations, amounting to about 2000 years, implies that the mutation has arisen in an ancestor living about 1,500 years before the arrival of the first Portuguese-Iberic settlers in Latin America. Furthermore, the fact that this 2000-year haplotype diversity is observed among Brazilians themselves implies that the current population of Brazilian p.Arg337His carriers originated from several distantly related ancestors who where separated by at least 50 generations (about 1000 years) at the time of their arrival on the Latin American continent. Indeed, assuming a 2000-years old history for the p.Arg337His haplotype, its geographic spread in Brazil cannot have taken place before the early 18th to mid-19th century, when the first large waves of migrants of Europeans settled in Southern Brazil. Finally, if the mutation is indeed European in origin, one would expect to observe it at more significant frequencies in European LFS/LFL families. Thus, a more detailed analysis of the common ancestry of p.Arg337His carriers, and perhaps an extended mutation search in other populations may be needed to understand the historical geographical spread of this alteration.

## Supporting Information

S1 Table DMLE Data. (XLSX)

S2 Table Arlequin Data. (XLS)
